# Afatinib induces pro-survival autophagy and increases sensitivity to apoptosis in stem-like HNSCC cells

**DOI:** 10.1038/s41419-021-04011-0

**Published:** 2021-07-22

**Authors:** Xianfang Liu, Huiyuan Suo, Shengli Zhou, Zhenxing Hou, Mingqiang Bu, Xiuxiu Liu, Wei Xu

**Affiliations:** 1 Department of Otolaryngology-Head and Neck Surgery, Shandong Provincial ENT Hospital, Cheeloo College of Medicine, Shandong University, Jinan, 250022 P.R. China; 2 Department of Otorhinolaryngology-Head and Neck Surgery, Tengzhou Central People’s Hospital, Tengzhou, Shandong 277500 P.R. China

**Keywords:** Apoptosis, Targeted therapies, Macroautophagy

## Abstract

Afatinib, a second-generation tyrosine kinase inhibitor (TKI), exerts its antitumor effects in head and neck squamous cell carcinoma (HNSCC) by inducing intrinsic apoptosis through suppression of mTORC1. However, the detailed mechanism and biological significance of afatinib-induced autophagy in HNSCC remains unclear. In the present study, we demonstrated that afatinib induced mTORC1 suppression-mediated autophagy in HNSCC cells. Further mechanistic investigation revealed that afatinib stimulated REDD1-TSC1 signaling, giving rise to mTORC1 inactivation and subsequent autophagy. Moreover, ROS generation elicited by afatinib was responsible for the induction of the REDD1-TSC1-mTORC1 axis. In addition, pharmacological or genetic inhibition of autophagy sensitized HNSCC cells to afatinib-induced apoptosis, demonstrating that afatinib activated pro-survival autophagy in HNSCC cells. Importantly, in vitro and in vivo assays showed that afatinib caused enhanced apoptosis but weaker autophagy in stem-like HNSCC cells constructed by CDH1 knockdown. This suggested that blocking autophagy has the potential to serve as a promising strategy to target HNSCC stem cells. In conclusion, our findings suggested that the combination treatment with afatinib and autophagy inhibitors has the potential to eradicate HNSCC cells, especially cancer stem cells in clinical therapy.

## Introduction

Head and neck squamous cell carcinoma (HNSCC) is one of the most common human malignancies, with a worldwide morbidity of more than 600,000 cases per year [1, 2]. Although obvious advances have been made in the clinical diagnosis and treatment of HNSCC, the 5-year survival rate has remained poor for the last few decades [3]. Therefore, there is an urgent need to develop effective therapy options to improve HNSCC survival rates.

Epidermal growth factor receptor (EGFR) is overexpressed in approximately 90% of HNSCC, and aberrant expression of EGFR is intimately connected with the dismal clinical outcomes of this disease [4]. As such, EGFR has become a focus for the development of targeted therapeutics, including anti-EGFR monoclonal antibody (mAb) and small molecule tyrosine kinase inhibitors (TKIs). However, first-generation EGFR-TKIs generated disappointing response rates in the treatment of HNSCC [5].

Afatinib, a second-generation TKI, is an oral irreversible ErbB family blocker that inhibits signaling from all ErbB family members [6]. In phase III trials (LUX-Head & Neck 1 and LUX-Head & Neck 3), afatinib has shown efficacy as a second-line treatment in patients with recurrent and/or metastatic (R/M) HNSCC [7, 8]. Moreover, many studies have worked to illuminate the molecular mechanism of afatinib-induced cell death. Afatinib was reported to function as an effective suppressor of ATP Binding Cassette Subfamily G Member 2 (ABCG2) and ABCB1, which provided a mechanism to circumvent multidrug resistance and eradicate cancer stem-like cells [9–11]. In nonsmall cell lung cancer (NSCLC) without EGFR mutation, afatinib was shown to induce apoptosis via Ets-like-1 protein (Elk-1)-mediated inhibition of cancerous inhibitor of PP2A (CIP2A) [12]. Moreover, in our previous studies, af﻿ati﻿nib trigg﻿ered intrinsic apoptosis of HNSCC cells through downregulation of MCL-1 via PERK-eIF2α-ATF4 signaling pathway [13]. In addition, recent studies revealed that stimulation of autophagy was involved in the induction of afatinib resistance in pancreatic ductal adenocarcinoma (PDAC). Furthermore, blocking autophagy via knockdown of Beclin-1 distinctly decreased the IC50 value of afatinib in resistant cells [14], and Xiangxiang Hu et al. demonstrated that afatinib caused autophagy in lung adenocarcinoma with activating EGFR mutations. Importantly, in vivo and in vitro assays demonstrated that suppression of autophagy with chloroquine (CQ) and 3-methyladenine (3-MA) markedly heightened afatinib-induced cytotoxicity, indicating that autophagy serves as a protective response in lung adenocarcinoma cells treated with afatinib [15]. Additionally, combined treatment with afatinib and suberoylanilide hydroxamic acid (SAHA), a histone deacetylase (HDAC) inhibitor, evoked proapoptotic autophagy in EGFR T790M-mutated lung cancer cells [16]. Co-treatment with afatinib also enhanced an adriamycin-induced antiproliferation effect in A549T cells, which may benefit from apoptosis and autophagy promoted by afatinib [17]. However, whether afatinib triggers autophagy in HNSCC cells remains to be determined.

Macroautophagy (hereafter referred to as autophagy) is a highly conserved cellular degradation process by which damaged proteins and organelles are encapsulated into autophagosome, and are then transported to lysosomes for digestion and recycling to sustain cellular homeostasis. In addition, autophagy can promote cell death in extreme physiological settings [18, 19]. Extensive research has revealed that autophagy can be modulated by a network of complex signaling pathways, including mammalian target of rapamycin (mTOR), phosphatidylinositol 3-kinase (PI3K), unfolded protein response (UPR), p53, and reactive oxygen species (ROS) [20, 21]. In the present study, we demonstrated that afatinib induced signaling of regulated in development and DNA damage responses 1 (REDD1)-tuberous sclerosis 1 (TSC1) through ROS generation, which ultimately leads to mTORC1 inactivation and subsequent autophagy in HNSCC cells. Furthermore, blocking autophagy made HNSCC cells more sensitive to afatinib-induced apoptosis. Importantly, afatinib triggered enhanced apoptosis but weaker autophagy in stem-like HNSCC cells constructed by CDH1 knockdown. Therefore, our study demonstrated for the first time that afatinib induced pro-survival autophagy in HNSCC cells. Furthermore, our findings suggested a novel approach for improving the clinical application of afatinib, by combining treatment with autophagy inhibitors in HNSCC therapy.

## Materials and methods

### Animals

Male BALB/c nude mice were purchased from Vital River Laboratories (Beijing, China). All animals were maintained on a 12-h light-dark cycle in the specified pathogen-free facility under standard feeding conditions at 23–27 °C and 60 ± 5% humidity. The animals had ad libitum access to water and food throughout the experiment.

### Cell lines and cell culture

Human HNSCC cell lines, FaDu and CAL-27 were initially obtained from the American Type Culture Collection (Manassas, VA). The HN6 cell line was a gift from Professor Wantao Chen. All cell lines were grown in monolayer culture in Dulbecco’s Modified Eagle Medium (DMEM)/F-12 (1:1) medium (C11330500BT, Gibco, Grand Island, NY) supplemented with 10% fetal bovine serum (FBS; 04-001-1ACS, BI, Israel) at 37 °C in a humidified atmosphere consisting of 5% CO_2_ and 95% air.

### Reagents

Afatinib, 3-MA, and rapamycin were obtained from Selleckchem (Houston, TX, USA); CQ was purchased from Sigma-Aldrich (St. Louis, MO, USA).

### Establishment of cell lines with stable overexpression of EGFP-LC3B

The EGFP-LC3B lentivirus was obtained from Vigene Biosciences (Shandong, China). FaDu and HN6 cells were infected with EGFP-LC3B lentivirus and then treated with puromycin for selection. Ultimately, pools were created of FaDu and HN6 cells with stable EGFP-LC3B overexpression.

### Establishment of cell lines with stable knockdown of CDH1

FaDu and HN6 cells expressing either control-RNAi (Ctrli) or CDH1-RNAi (CDH1i) were created by infecting cells with GV248-control-RNAi and GV248-CDH1-RNAi lentivirus, respectively. The lentiviruses were obtained from Genechem (Shanghai, China). Stable transfectants of FaDu-Ctrli/FaDu-CDH1i and HN6-Ctrli/HN6-CDH1i were selected with puromycin and pooled.

### Western blot analysis

Preparation of whole-cell protein lysates and western blot analysis were carried out as previously described [22]. Briefly, cells were lysed in lysis buffer containing 1× protease inhibitor cocktail and 1× phosphatase cocktail. The proteins in cell lysates were then separated using SDS-PAGE and protein bands were transferred to polyvinylidene difluoride (PVDF) membranes (Bio-Rad, Hercules, CA, USA) by electroblotting. The membranes were incubated with appropriate primary antibodies overnight at 4 °C, and incubated with the secondary antibodies for 1 h, and then the protein bands were detected by the ECL system (MERK, New Jersey, USA), according to the manufacturer’s protocol. The primary antibodies used in this study are as follows: LC3B (Cat# 2775S), Beclin-1 (Cat# 3738S), TSC1 (Cat# 6935S), TSC2 (Cat# 4308S), PARP (Cat# 9542S), REDD1 (Cat# 2516S), FN1 (Cat# 26836S), N-cad (Cat# 13116S), SOX2 (Cat# 14962S), and Oct4 (Cat# 2750S) antibodies were purchased from Cell Signaling Technology (Danvers, MA, USA). P62 (Cat# 610832) and CDH1 (Cat# 610181) antibodies were purchased from BD Biosciences (New Jersey, USA); Caspase-3 (Cat# NB100-56708) antibody was purchased from Novus Biologicals (CO, USA); Atg5 (Cat# ab108327), mTOR (phosphor S2448) (Cat# ab109268), and Twist (Cat# ab175430) antibodies were purchased from Abcam (Cambridge, MA, USA); and β-actin (Cat# TA-09) antibody was obtained from ZSJB-BIO (Beijing, China).

### Transfection of small interfering RNAs

Small interfering RNAs (siRNAs) were synthesized by GenePharma (Shanghai, China). Atg5 siRNA duplexes target the sequence of 5′-CCTTTGGCCTAAGAAGAAA-3′; REDD1 siRNA duplexes target the sequence of 5′-GTGGAGACTAGAGGCAGGAGC-3′; and TSC1 and TSC2 siRNA duplexes target the sequences of 5′-AAACACGTTGGTGAATTATTA-3′ and 5′-CAAUGAGUCACAGUCCUUUGA-3′, respectively. Control siRNA duplexes target the sequence of 5′-UUCUCCGAACGUGUCACGU-3′. The cells were transfected with siRNAs using Lipofectamine^®^ RNAi MAX Reagent (Invitrogen, CA, United States) according to the manufacturer’s instructions. Western blot assays were performed to assess gene knockdown.

### Cell death detection and IC_50_

The cell survival was assessed using the Sulforhodamine B (SRB) assay, as previously described [13]. The cells were seeded in 96-well plates and treated with the indicated concentration of afatinib for 24 h on the second day. Followed by the treatment medium was discarded, the cells were fixed with 100 μl cold trichloroacetic acid (10% (w/v)) at 4 °C for 1 h. Then, the plates were washed five times with deionized water and air-dried. Subsequently, 50 μl 0.4% (w/v) SRB solution (dissolved in 1% acetic acid) was added into each well for 5 min at room temperature. The plates were then washed with 1% acetic acid for five times to remove uncombined SRB. The combined SRB was dissolved with 100 μl 10 mM Tris base buffer (pH 10.5) and absorbance was measured at 495 nm with a microtiter plate reader (BioTek, USA). Absorbency was considered to be positively connected with cell survival. In addition, apoptosis was evaluated using an Annexin V-FITC/PI Apoptosis Kit obtained from Lianke Sciences (Hangzhou, China) and the manufacturer’s protocol was followed. Caspase activation was examined by western blot analysis. According to the results of the cell survival assay in our previous study^13^, the IC_50_ value of afatinib in FaDu, HN6, and CAL-27 cell lines was calculated using IBM SPSS Statistics software (Version 25).

### Tumor xenograft model

FaDu-Ctrli and FaDu-CDH1i cells (2 × 10^6^) were inoculated subcutaneously into the right flanks of 5-week-old BALB/c nude mice in a mixture of 80 μL 1× PBS and 20 μL Matrigel™ (BD Biosciences, New Jersey, USA). When the average tumor volume reached 200 mm [3], the nude mice were randomly divided into two groups in each xenograft model (five mice per group), and the mice then received lavage administration of afatinib (10 mg/kg) or vehicle every 2 days. Tumor volume was measured every other day and calculated according to the following formula: V = (length × width^2^)/2. After treatment for 20 days, the animals were sacrificed and the tumors were stripped, followed by paraformaldehyde fixation for immunohistochemistry analysis. The tumor growth curve was plotted with treatment day as the horizontal axis and tumor volume as the vertical axis.

### Immunohistochemistry

Tumors stripped from the xenograft model were paraffin embedded and cut to 2 μm sections. The sections were then stained for CDH1, SOX2, Ki67, LC3B, and cleaved caspase-3 using a Biotin-Streptavidin HRP Detection System, according to the manufacturer’s instructions (ZSJB-BIO, Beijing, China). Briefly, histological sections were deparaffinized and rehydrated, and then antigen retrieval was performed in a water bath kettle at 95 °C for 20 min. After treating with endogenous peroxidase blockers for 13 min, the sections were incubated with goat serum working solution for 15 min to avoid nonspecific binding, and were then incubated with rabbit anti-CDH1 (Cat# ZA0565, ZSJB-BIO), SOX2 (Cat# 14962S, Cell Signaling Technology), Ki67 (Cat# ZA-0502, ZSJB-BIO), LC3B (Cat# 3868S, Cell Signaling Technology), or cleaved caspase-3 (Cat# 9664S, Cell Signaling Technology) primary antibody at 4 °C overnight. Following incubation with HRP-conjugated goat antirabbit secondary antibody, 3,3′-diaminobenzidine tetrachloride (DAB) was applied to detect HRP activity. All histopathological pictures were captured with an Olympus BX53 microscope (Nagano, Japan).

### Measurement of ROS

The cellular ROS accumulation was measured with an ROS detection kit (Beyotime Biotechnology, Shanghai, China), according to the manufacturer’s instructions. In brief, the cells were seeded in 6-cm culture dishes, treated with 2 μM afatinib for 24 h, and were then washed twice with 1× PBS. Next, 2 × 10^5^ cells were collected and incubated with 10 μM DCFH-DA for 20 min. The cell pellet was suspended with 500 μL 1× PBS and cytometry analysis was performed to detect fluorescent signal intensity with excitation at 488 nm and emission at 525 nm. Flow cytometry data were analyzed using Cell Quest software.

### Cell proliferation assay

Cell proliferation was assessed using a CCK8 kit (Beyotime Biotechnology, Shanghai, China). FaDu-Ctrli/FaDu-CDH1i and HN6-Ctrli/HN6-CDH1i cells were seeded in 96-well plates and incubated for 24 h, 48 h, and 72 h. Thereafter, 10 μL CCK8 solution was added to each well and incubated for 2 h at 37 °C. Absorbance was measured at 450 nm using a microtiter plate reader (BioTek, USA).

### EdU staining

A 5-ethynyl-2′-deoxyuridine (EdU) incorporation assay was conducted using a kFluor647 Click-iT EdU Imaging Test Kit (KeyGen BioTec, Nanjing, China), according to the manufacturer’s protocol. Briefly, FaDu-Ctrli/FaDu-CDH1i or HN6-Ctrli/HN6-CDH1i cells were incubated with DMEM/F-12 medium containing 10 μM EdU for 2 h. Next, the cells were fixed with 4% paraformaldehyde for 30 min, neutralized with 2 mg/mL glycine for 5 min, and then permeabilized with 0.5% Triton X-100 solution. After washing with 1× PBS, the cells were incubated with the Click-iT reaction mixture for 30 min at room temperature. In addition, DAPI that binds to the DNA double strands was added to label nuclei. Finally, the images of the cells were photographed utilizing a laser scanning confocal microscope (Leica, Germany). The proportion of EdU-positive cells compared to DAPI-labeled cells was determined to evaluate the proliferation capacity of the tested cells.

### Invasion and migration assay

Transwell™ chambers (8 μm, 24-well format; Costar, MA, USA) were coated with or without Matrigel (BD Biosciences, NY, USA) diluted 1:3 with serum-free DMEM/F-12 medium to conduct invasion and migration assays. In short, FaDu-Ctrli/FaDu-CDH1i (1 × 10^5^ cells for invasion and migration assays) and HN6-Ctrli/HN6-CDH1i cells (3 × 10^4^ cells for invasion assay and 1 × 10^4^ cells for migration assay) were suspended in 250 μL serum-free DMEM/F-12 medium. The cells were seeded in the upper chamber, and 750 µL DMEM/F-12 containing 20% FBS was added to the lower chamber. After incubating for 24 h, cells on the upper membrane were wiped off; cells that invaded or migrated under the membrane were fixed with 4% paraformaldehyde and stained with Crystal Violet. Four random fields were captured with a microscope, and stained cells were counted. Each assay was performed in triplicate.

### Real-time quantitative PCR (RT-qPCR)

Total RNA was extracted using a Direct-zol^TM^ RNA MiniPrep Kit (Zymto Research, CA, USA). Reverse transcription-polymerase chain reaction (RT-PCR) was conducted using a RevertAid First Strand cDNA Synthesis Kit (ThermoFisher Scientific, Waltham, MA, USA) and subsequent real-time quantitative PCR was performed using a TB Green^®^ Premix Ex Taq^TM^ (Tli RNaseH plus) Kit (TaKaRa Biology (Dalian), Liaoning, China). β-actin was used as an internal control. The primers used in this study are listed in Supplementary Table 1.

### Statistical analysis

Statistical analysis was conducted with GraphPad Prism 5.0 and SPSS 17.0 software. Differences between two groups were analyzed with two-sided unpaired Student’s *t* tests; differences across multigroups were analyzed with one-way ANOVA, followed by the least-significant difference post hoc test or two-way ANOVA. The tumor volume data included many repeated measurements, and therefore, differences in tumor volume between the two groups were assessed using multivariate analysis of variance (MANOVA). The data were deemed to be statistically significant when the *P-*value was less than 0.05.

## Results

### Afatinib induces autophagy in HNSCC cells

**Fig. 1:**
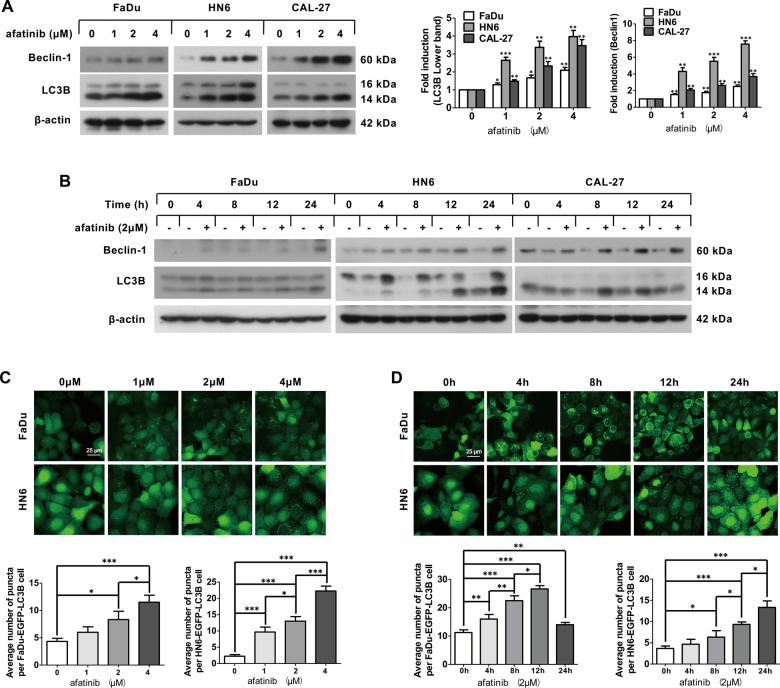
Afatinib induces autophagy in HNSCC cells. **A** Western blot analysis of Beclin-1 and LC3B expression in FaDu, HN6, and CAL-27 cells after treatment with 0, 1, 2, and 4 μM afatinib for 24 h. The intensity of Beclin-1 and LC3B was normalized to β-actin, and the fold induction (treated/untreated control) was calculated (*p < 0.05, **p < 0.01, ***p < 0.001 vs. untreated control). **B** Western blot analysis of Beclin-1 and LC3B expression in FaDu, HN6, and CAL-27 cells after treatment with 2 μM afatinib for 0, 4, 8, 12, and 24 h. **C**, **D** FaDu-EGFP-LC3B and HN6-EGFP-L**C**3B cell lines were treated with 0, 1, 2, and 4 μM afatinib for 24 h (**C**) or treated with 2 μM afatinib for 0, 4, 8, 12, and 24 h (**D**). Then, cells were fixed, imaged, and EGFP-LC3B fluorescent spots were quantified. Scale bar, 25 µm. All data are presented as the mean ± SD from three independent experiments. *p < 0.05, **p < 0.01, ***p < 0.001.

To determine whether afatinib induces autophagy in HNSCC, we examined the transformation of LC3B-II that was converted from LC3B-I by conjugating with phosphatidylethanolamine, which is regarded as a marker of autophagy. According to the IC_50_ value of afatinib in FaDu, HN6, and CAL-27 cell lines (Supplementary Table 2), we chose the concentration of 0, 1, 2, and 4 μM to carry out the dose assays. After treating with the indicated concentrations of afatinib for 24 h or exposing to 2 μM afatinib for the indicated time, there was a marked increase in the conversion of LC3B-II in both a dose- and a time-dependent fashion (Fig. 1A, B). Consistently, the expression of Beclin-1, a constituent of the class III PI3K complex, was also upregulated following afatinib treatment in a dose- and a time-dependent manner (Fig. 1A, B).

To further monitor autophagosome formation, we established FaDu-EGFP-LC3B and HN6-EGFP-LC3B cell lines that stably expressed the EGFP-LC3B fusion gene. After exposing to the indicated concentration of afatinib for 24 h, the average EGFP-LC3B fluorescence per cell was elevated in a dose-dependent manner in FaDu-EGFP-LC3B and HN6-EGFP-LC3B cell lines (Fig. 1C). Meanwhile, we also observed a time-dependent increase in EGFP-LC3B puncta per cell in FaDu-EGFP-LC3B and HN6-EGFP-LC3B cell lines following treatment with 2 μM afatinib for the indicated time (Fig. 1D).

### Afatinib triggers autophagic flux in human HNSCC cells

**Fig. 2:**
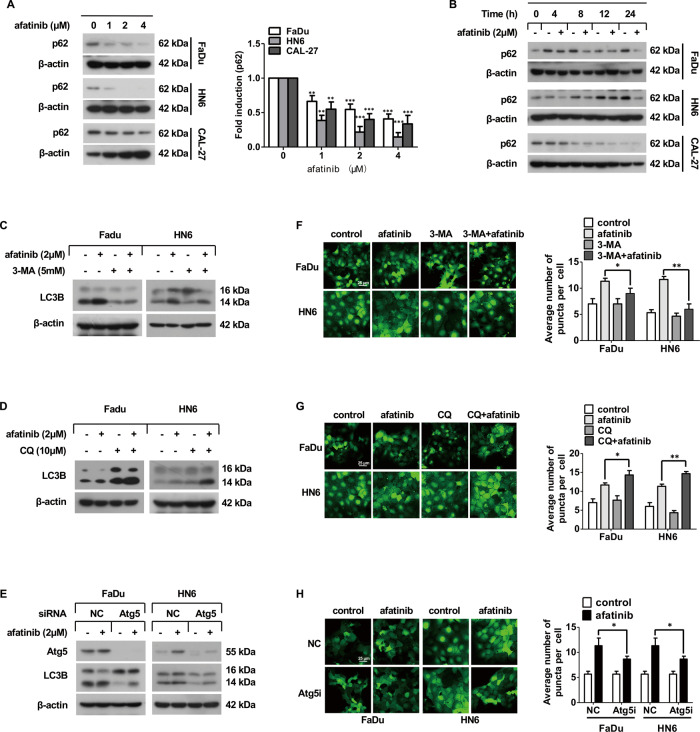
Afatinib triggers autophagic flux in human HNSCC cells. **A** Western blot analysis of p62 expression in FaDu, HN6, and CAL-27 cells after treatment with 0, 1, 2, and 4 μM afatinib for 24 h. The intensity of p62 was normalized to β-actin, then the fold induction (treated/untreated control) was calculated (**p < 0.01, ***p < 0.001 vs. untreated control). **B** Western blot analysis of p62 expression in FaDu, HN6, and CAL-27 cells after treatment with 2 μM afatinib for 0, 4, 8, 12, and 24 h. **C**, **D** FaDu and HN6 cells were pretreated with 5 mM 3-MA for 2 h (**C**) or with 10 μM CQ for 30 min (**D**), and then co-incubated with 2 μM afatinib for another 24 h. The expression of LC3B was detected by western blot analysis. **E** FaDu and HN6 cells were seeded in 6-well plates and transfected with control and Atg5 siRNA on the second day. After 48 h transfection, cells were exposed to 2 μM afatinib for 24 h. The expression of Atg5 and LC3B was detected by western blot analysis. **F**, **G** FaDu-EGFP-LC3B and HN6-EGFP-LC3B cells were pretreated with 5 mM 3-MA for 2 h (**F**) or with 10 μM CQ for 30 min (**G**), and then co-incubated with 2 μM afatinib for another 24 h. Thereafter, cells were fixed, imaged, and EGFP-LC3B fluorescent spots were quantified. **H** FaDu-EGFP-LC3B and HN6-EGFP-LC3B cells were transfected with control and Atg5 siRNA for 48 h. Then, cells were exposed to 2 μM afatinib for 24 h and the EGFP-LC3B fluorescent spots were determined using a laser scanning confocal microscope. Scale bar, 25 µm. All data are presented as the mean ± SD from three independent experiments. *p < 0.05, **p < 0.01.

To monitor the autophagic flux, we measured p62, the specific substrate for autophagy degradation. The dose- and time-dependent western blot results demonstrated that afatinib markedly downregulated p62 expression in FaDu, HN6, and CAL-27 cell lines (Fig. 2A, B). In addition, autophagy inhibitors were applied to assess the alteration of autophagic flux. As shown in Fig. 2C, F, co-incubation with afatinib and 3-MA, an upstream suppressor of autophagy, significantly decreased the level of LC3B-II, as well as the number of EGFP-LC3B puncta. In contrast, co-incubation with afatinib and CQ, which blocks the downstream of autophagy, markedly increased the transformation of LC3B-II (Fig. 2D) and the number of EGFP-LC3B puncta (Fig. 2G). Furthermore, siRNA knockdown of Atg5 expression in FaDu and HN6 cells markedly reduced LC3B-II (Fig. 2E) and EGFP-LC3B puncta transformation (Fig. 2H). Collectively, these data suggested that afatinib strongly stimulated autophagy in human HNSCC cells.

### Afatinib causes mTORC1-mediated autophagy via TSC1 but not TSC2

**Fig. 3:**
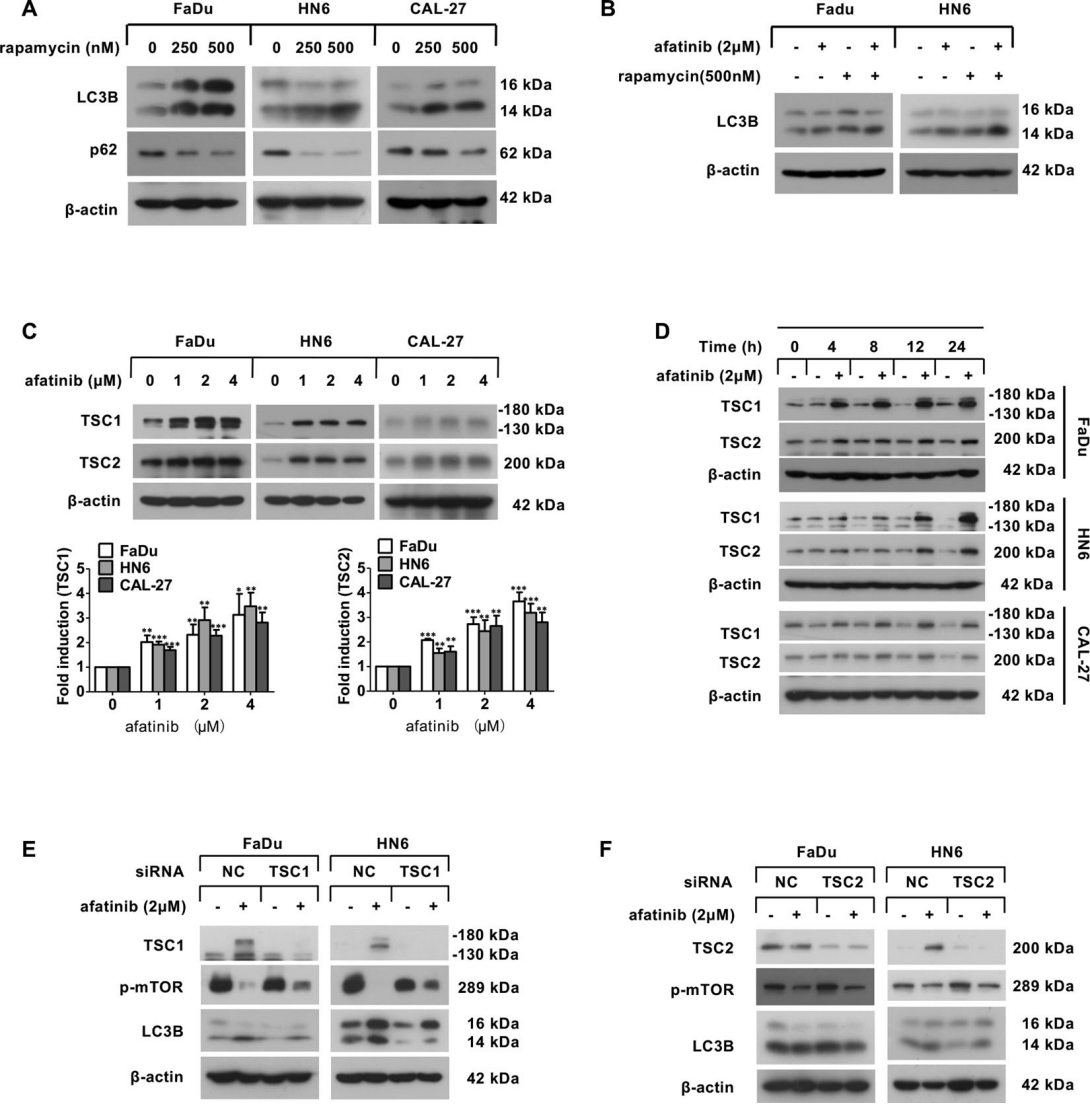
Afatinib upregulates TSC1, but not TSC2, to contribute to mTOR-mediated autophagy. **A** FaDu, HN6, and CAL-27 cells were treated with 0, 250, 500 nM rapamycin for 24 h, and then the expression of LCB and p62 was analyzed by western blot analysis. **B** FaDu and HN6 cells were pretreated with 500 nM rapamycin for 30 min followed by co-incubation with 2 μM afatinib for another 24 h, and then western blot analysis was performed to examine the level of LC3B. **C** Western blot analysis of TSC1 and TSC2 expression in FaDu, HN6, and CAL-27 cells after treatment with 0, 1, 2, and 4 μM afatinib for 24 h. The intensity of TSC1 and TSC2 was normalized to β-actin, then the fold induction (treated/untreated control) was calculated (**p < 0.01, ***p < 0.001 vs. untreated control). **D** Western blot analysis of TSC1 and TSC2 expression in FaDu, HN6, and CAL-27 cells after treatment with 2 μM afatinib for 0, 4, 8, 12, and 24 h. **E**, **F** FaDu and HN6 cells were transfected with TSC1 siRNA (**E**) or TSC2 siRNA (**F**). Forty-eight hours after transfection, cells were treated with 2 μM afatinib for 24 h. The level of TSC1, TSC2, p-mTOR, and LC3B was detected by western blot analysis.

It is well established that mTORC1 negatively regulates autophagy [23], and our previous study revealed that afatinib inhibits mTORC1 activity [13]. Therefore, we next wanted to determine whether mTORC1 played a vital role in afatinib-induced autophagy. To answer this question, we used rapamycin, an inhibitor of mTORC1, to imitate the effect of mTOR suppression in HNSCC cell lines. After rapamycin treatment, LC3B-II conversion was obviously increased, whereas p62 was dramatically decreased in a dose-dependent manner (Fig. 3A). Next, we pretreated FaDu and HN6 cells with rapamycin for 30 min before incubating with afatinib for another 24 h, and found that co-incubation with afatinib and rapamycin elicited an enhanced conversion of LC3B-II (Fig. 3B). Thus, these data demonstrated that afatinib induced autophagy via inactivation of mTORC1.

Based on existing results, we further inspected the molecular differences in three HNSCC cell lines by western blot analysis and found that basal expression of Beclin-1 and LC3B-II was stronger and expression of p62 was lower in CAL-27 cells compared to FaDu or HN6 cells (Supplementary Table 3). In addition, mTOR phosphorylation was greatest in CAL-27 cells (Supplementary Table 3). These results indicated that CAL-27 cells had the highest basal autophagy level. Meanwhile, the autophagic response to afatinib treatment in CAL-27 cells may be less sensitive than that in FaDu or HN6 cells due to higher p-mTOR expression; this was partially accounted for the difference in afatinib-induced autophagy between FaDu, HN6, and CAL-27 cell lines (Fig. 1B). Moreover, these data further suggested that mTOR inactivation mediated afatinib-induced autophagy in HNSCC cells.

Because previous studies have reported that TSC1/2 acts as a negative regulator of mTORC1 [24], we hypothesized that upregulation of TSC1/2 might be involved in mTOR inactivation-mediated autophagy with afatinib. Indeed, the expression of TSC1 and TSC2 was upregulated in a dose- and a time-dependent manner in HNSCC cells following treatment with afatinib (Fig. 3C, D). To further ascertain whether TSC1 and TSC2 were required for mTOR-mediated autophagy, we used siRNA to silence TSC1 or TSC2 in FaDu and HN6 cells. We found that silence of TSC1 (Fig. 3E), but not TSC2 (Fig. 3F), partially abolished the mTOR inactivation and LC3B-II conversion that was caused by afatinib. Therefore, these data suggested that the afatinib-induced autophagy that attributed to mTOR inactivation in HNSCC cells was likely mediated by TSC1, and not TSC2.

### ROS-dependent upregulation of REDD1 contributes to afatinib-induced autophagy

**Fig. 4:**
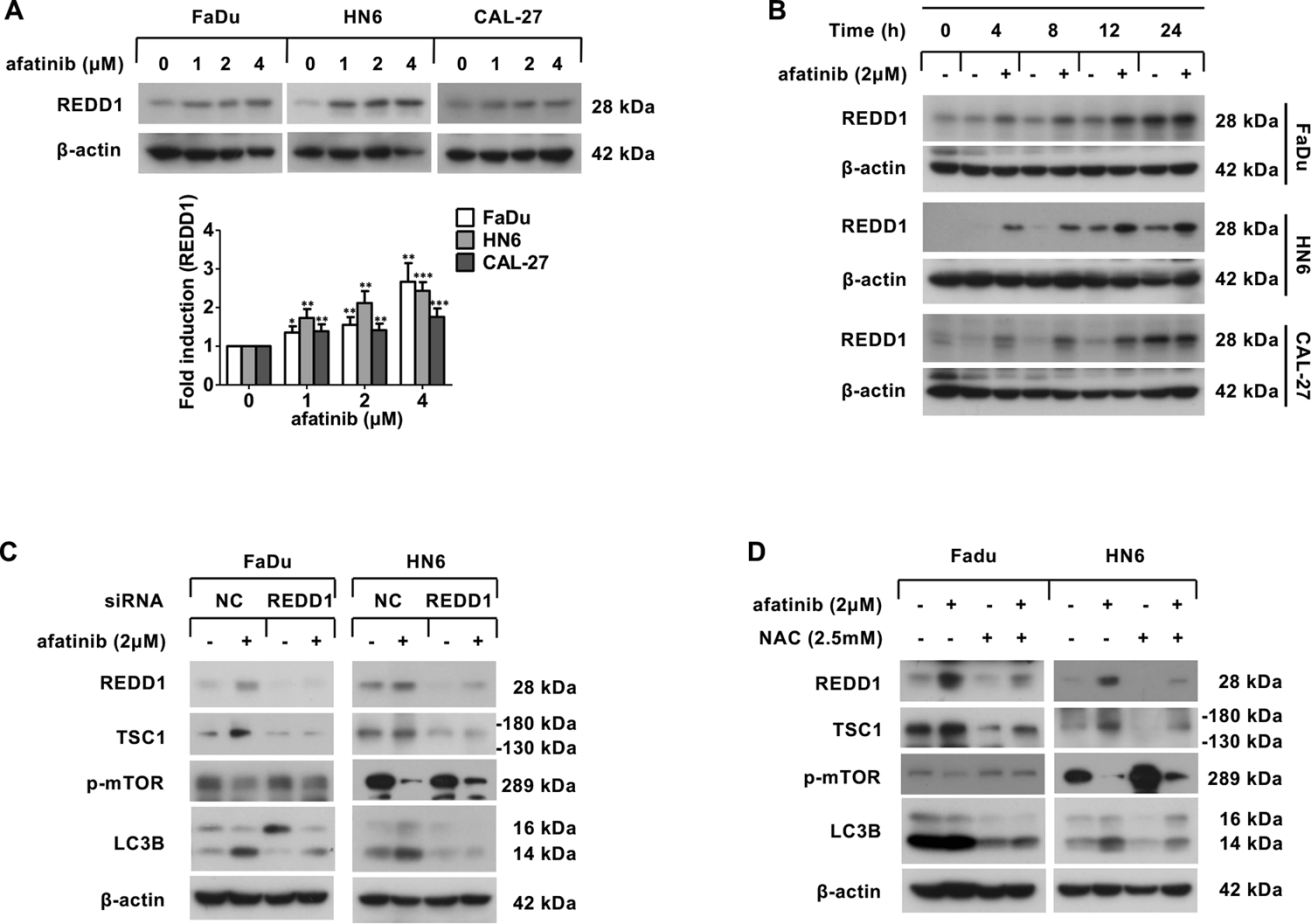
Afatinib induces autophagy through ROS-dependent upregulation of REDD1. **A** Western blot analysis of REDD1 expression in FaDu, HN6, and CAL-27 cells after treatment with 0, 1, 2, and 4 μM afatinib for 24 h. The intensity of REDD1 was normalized to β-actin, then the fold induction (treated/untreated control) was calculated (*p < 0.05, **p < 0.01, ***p < 0.001 vs. untreated control). **B** Western blot analysis of REDD1 expression in FaDu, HN6, and CAL-27 cells after treatment with 2 μM afatinib for 0, 4, 8, 12, and 24 h. **C** FaDu and HN6 cells were transfected with control and REDD1 siRNA. Forty-eight hours later, cells were incubated with 2 μM afatinib for 24 h. The level of REDD1, TSC1, p-mTOR, and LC3B was measured by western blot analysis. **D** FaDu and HN6 cells were pretreated with 2.5 mM NAC for 30 min followed by co-incubation with 2 μM afatinib for another 24 h, and then the level of REDD1, TSC1, p-mTOR, and LC3B was measured by western blot analysis.

It has been documented that REDD1-TSC1 signaling is essential for the suppression of mTORC1 [25, 26]. Therefore, we next asked whether afatinib-induced activation of the TSC1-mTOR axis was attributed to the upregulation of REDD1. As shown in Fig. 4A, B, the expression of REDD1 was significantly upregulated in both a dose- and a time-dependent fashion following afatinib treatment. Moreover, siRNA silencing of REDD1 expression attenuated the upregulation of TSC1 as well as the inactivation of mTOR, which in turn reduced the conversion of LC3B-II following afatinib treatment (Fig. 4C). Therefore, these findings demonstrated that the upregulation of REDD1 mediated TSC1-mTOR signaling and the subsequent autophagy response that was induced by afatinib treatment.

Because ROS have been reported to modulate REDD1 expression and also promote autophagy [27, 28], we next questioned whether ROS were responsible for afatinib-induced autophagy that was mediated by REDD1. ROS detection by dichlorofluorescein diacetate (DCFH-DA) staining revealed an increase in ROS levels following afatinib treatment (Fig. S1A, B). Moreover, preincubation with N-acetyl cysteine (NAC) effectively halted afatinib-induced upregulation of REDD1 and TSC1, inactivation of mTOR, and conversion of LC3B-II (Fig. 4D). Taken together, these results supported the hypothesis that afatinib-generated ROS activated the REDD1-TSC1-mTORC1 axis and subsequent autophagy in HNSCC cells.

### Afatinib-induced autophagy plays a pro-survival role in HNSCC

**Fig. 5:**
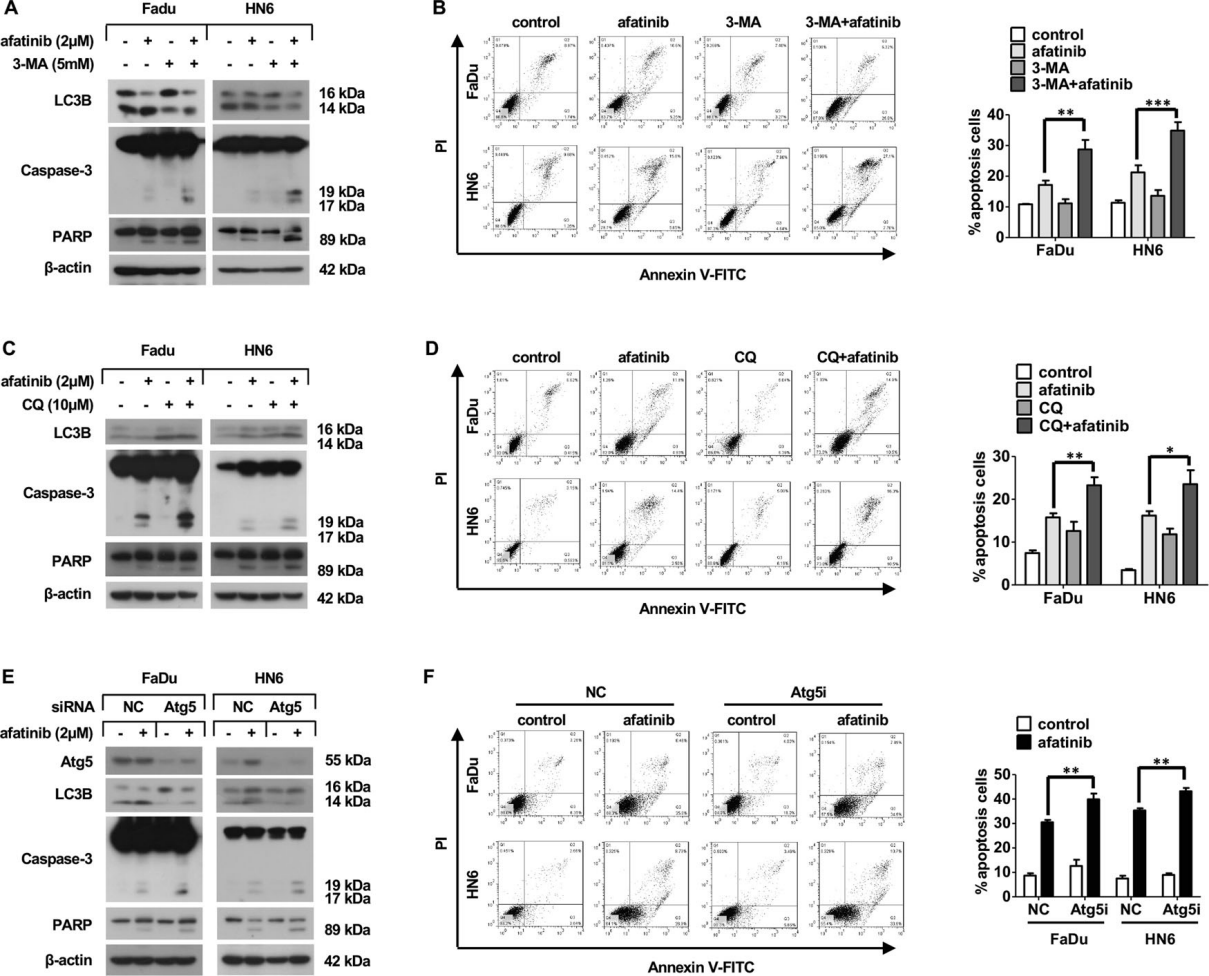
Afatinib-triggered autophagy plays a protective role in HNSCC. **A–D** FaDu and HN6 cells were pretreated with 5 mM 3-MA for 2 h (**A**, **B**) or with 10 μM CQ for 30 min (**C**, **D**) followed by co-treatment with 2 μM afatinib for another 24 h. The level of LC3B and apoptosis-associated proteins caspase-3 and PARP was measured by western blot analysis (**A**, **C**), while flow cytometry analysis was carried out to evaluate apoptosis (**B**, **D**). **E**, **F** FaDu and HN6 cells were transfected with control and Atg5 siRNA. Forty-eight hours after transfection, cells were exposed to 2 μM afatinib for 24 h. Atg5, LC3B, and apoptosis-associated proteins caspase-3 and PARP were measured by western blot analysis (**E**), meanwhile flow cytometry analysis was carried out to evaluate apoptosis (**F**). All data are presented as mean ± SD from three independent experiments. *p < 0.05, **p < 0.01, ***p < 0.001.

Because autophagic cell fate can differ depending on physiological circumstances [29], we next explored the function of afatinib-induced autophagy in HNSCC. We inhibited autophagy by co-incubating cells with autophagy inhibitors (3-MA or CQ) and afatinib, and we observed an increase in the cleaved forms of caspase-3 and PARP (Fig. 5A, C). Correspondingly, flow cytometry analysis also showed a pronounced increase of afatinib-induced apoptosis when cells were co-incubated with autophagy inhibitors (Fig. 5B, D). Furthermore, siRNA knockdown of Atg5 resulted in enhanced cleavage of caspase-3 and PARP (Fig. 5E). Moreover, flow cytometry analysis showed a notable enhancement of apoptosis in cells following Atg5 knockdown (Fig. 5F). Together, these results provided compelling evidence that afatinib-induced autophagy played a protective role in HNSCC cells.

### HNSCC cells with CDH1 depletion are more sensitive to afatinib treatment in vitro

Growing evidence has demonstrated that loss of CDH1 expression triggers epithelial-mesenchymal transition (EMT), which endows cancer cells with stem-like traits [30–32]. In addition, afatinib has been shown to reinforce radiosensitivity and chemosensitivity by eliminating cancer stem cells [10, 33]. Therefore, we speculated that afatinib might evoke severe cell death in parallel with weaker autophagy in HNSCC cells with CDH1 depletion. First, we detected the expression of the putative mesenchymal stem marker CD44 to evaluate the proportion of stem cells in FaDu and HN6 cell lines. As shown in Fig. S2A and 2B, positive CD44 staining in FaDu and HN6 cell lines was (23.9 ± 0.02)% and (27.1 ± 0.03)%, respectively. Next, we infected FaDu and HN6 cells with CDH1i lentivirus to establish FaDu-CDH1i and HN6-CDH1i cells with stable knockdown of CDH1 expression. FaDu-Ctrli and HN6-Ctrli cells infected with Ctrli lentivirus served as the corresponding control. In line with previous studies, FaDu and HN6 cells with CDH1 knockdown harbored enhanced capacity for cell growth, proliferation, migration, and invasion compared with control cells (Fig. S3A–E). But there was no difference in ROS level between CDH1-depleted cells and control cells (Fig. S4A, B). Moreover, in these cells, we observed significant upregulation of mesenchymal markers FN1, N-cad, and Twist (Fig. S3F, G). SOX2 and Oct4 are well-known hallmarks of stem cells, and both were markedly upregulated in cells with stable CDH1 knockdown (Fig. S3F, G). Thus, these established cell lines were used to represent cancer stem-like cells for follow-up experiments.

**Fig. 6:**
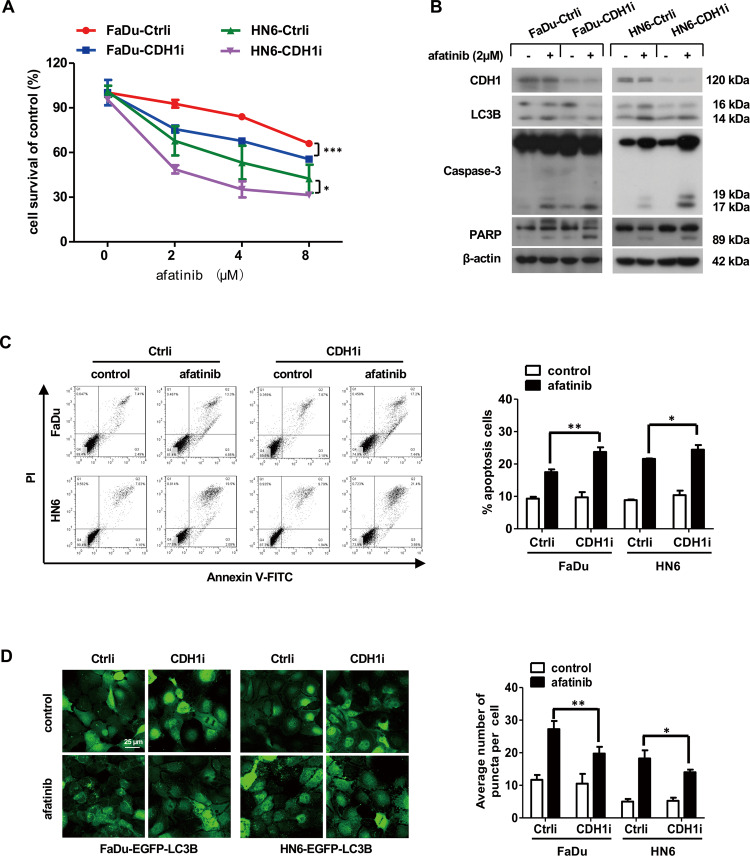
Afatinib induces more apoptosis and less autophagy in HNSCC cells with CDH1 depletion. **A** FaDu and HN6 cells with CDH1 depletion as well as the corresponding control cells were treated with the indicated concentration of afatinib for 24 h. Cell viability was examined by SRB assay. Statistical differences between the two groups were evaluated by two-way ANOVA. *p < 0.05, ***p < 0.001. **B**, **C** FaDu and HN6 cells with CDH1 depletion as well as control cells were treated with 2 μM afatinib for 24 h. The levels of CDH1, LC3B, and apoptosis-associated proteins caspase-3 and PARP were measured by western blot analysis (**B**), while flow cytometry analysis was carried out to evaluate apoptosis (**C**). **D** FaDu and HN6 cells stably expressing EGFP-LC3B were treated with 2 μM afatinib for 24 h. Cells were then fixed, imaged, and EGFP-LC3B fluorescent spots were quantified. Scale bar, 25 µm. The data are presented as mean ± SD from three independent experiments. *p < 0.05, **p < 0.01, ***p < 0.001.

To determine whether afatinib exhibited differing effects on stem-like HNSCC cells, we treated the indicated cell lines with various concentrations of afatinib for 24 h and examined cell survival by SRB assay. Following afatinib incubation, CDH1-depleted HNSCC cells had reduced viability compared to control cells (Fig. 6A). To ascertain whether afatinib triggered a stronger apoptotic response in CDH1-depleted cells, we further detected apoptosis-related proteins by western blot analysis and found increased expression of cleaved forms of caspase-3 and PARP in CDH1-depleted cells compared to control cells following afatinib treatment (Fig. 6B). Meanwhile, flow cytometry analysis provided further evidence that afatinib induced more apoptosis in HNSCC cells with CDH1 depletion (Fig. 6C). Together, these data demonstrated that afatinib enhanced sensitivity to apoptosis in stem-like HNSCC cells.

With the knowledge that afatinib-induced protective autophagy in HNSCC cells, we further examined whether afatinib triggered less autophagy in CDH1-depleted cells. As expected, afatinib treatment induced less conversion of LC3B-II in CDH1-depleted cells compared to control cells (Fig. 6B). To monitor the difference of LC3B fluorescent spot formation, we performed a secondary infection in FaDu-EGFP-LC3B and HN6-EGFP-LC3B cells with CDH1i lentivirus to stably knockdown CDH1 expression; control cells were infected with Ctrli lentivirus. Consistently, the expression of mesenchymal markers (FN1, N-cad, Twist) and stem cell markers (SOX2, Oct4) was greatly upregulated in CDH1-depleted cells (Fig. S5A, B). When compared to control cells, less aggregation of EGFP-LC3B fluorescent spots was observed in CDH1-depleted cells following afatinib treatment (Fig. 6D). Therefore, these in vitro data implied that a weaker autophagic response to afatinib in stem-like HNSCC cells may have accounted, to some extent, for the stronger apoptotic response.

### Afatinib enhances the suppression of FaDu-CDH1i xenograft tumor growth in vivo

**Fig. 7:**
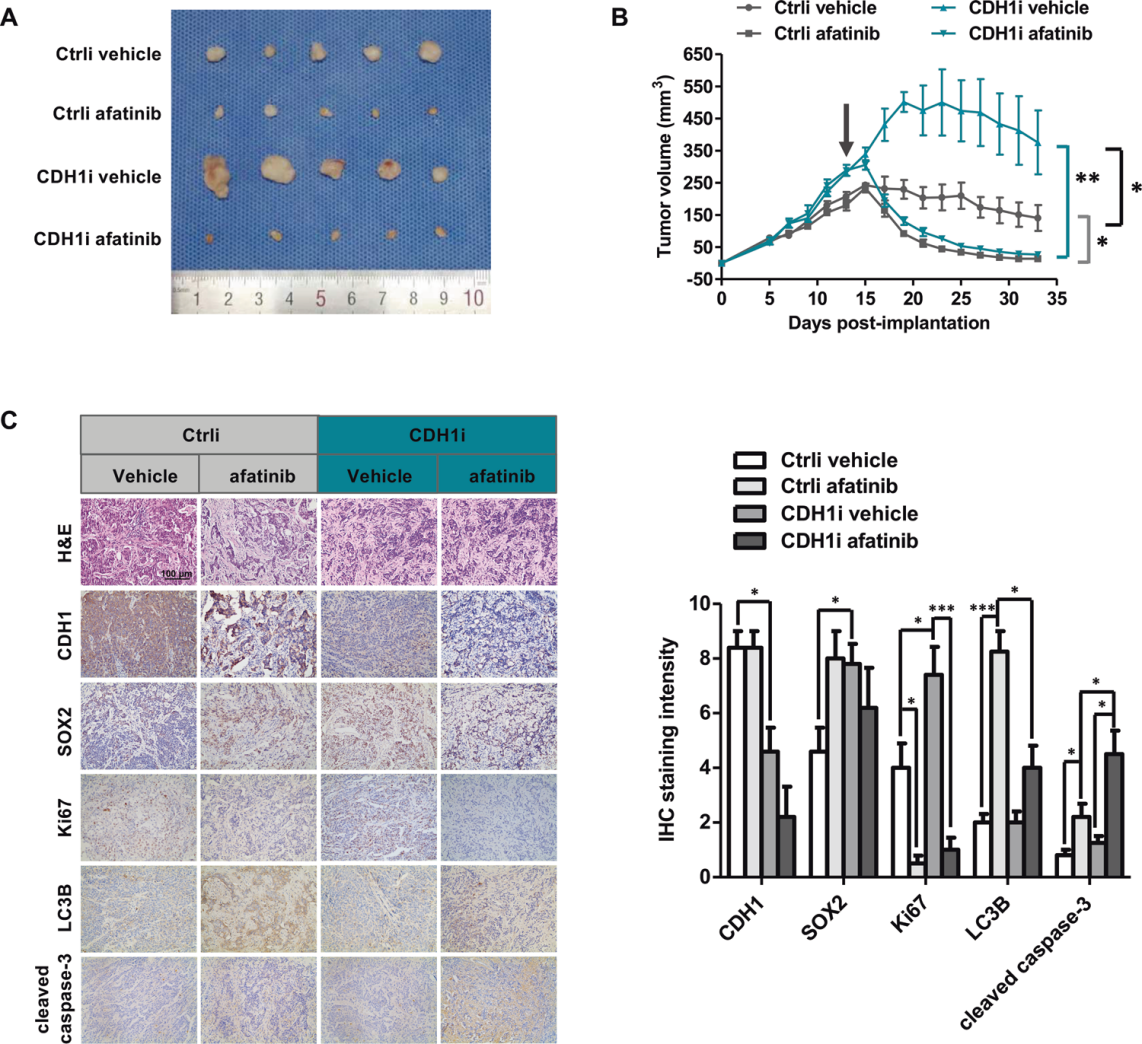
Afatinib effectively inhibits FaDu-CDH1i xenograft tumor growth in vivo. **A** The two established xenograft athymic nude mouse models that carried FaDu-Ctrli and FaDu-CDH1i tumors were treated with vehicle and afatinib (10 mg/kg) in each group. Nude mice were sacrificed after 20 days of treatment, and tumors were dissected and imaged. **B** Tumor growth was measured every other day. The black arrow indicates the initial time of treatment. **C** Immunohistochemistry staining for CDH1, SOX2, Ki67, LC3B, and cleaved caspase-3 was performed in serial paraffin sections from xenograft tumors. Scale bar, 100 µm. All data are presented as mean ± SEM. *p < 0.05, **p < 0.01, ***p < 0.001.

To evaluate whether tumor growth of FaDu-CDH1i cells was more intensely inhibited by afatinib, FaDu-CDH1i and FaDu-Ctrli cells were subcutaneously injected into the right flanks of nude mice to create stem-like xenograft and control xenograft mouse models. When tumor volumes reached approximately 200 mm^3^, each xenograft model was randomly assigned to the vehicle control group or the afatinib treatment group. As shown in Fig. 7A, B, afatinib treatment resulted in distinct tumor regression in both xenograft models. Strikingly, the afatinib-induced tumor shrinkage was more pronounced in the FaDu-CDH1i xenograft model than in the control xenograft model (Fig. 7B). Immunohistochemistry was performed to detect the expression of CDH1, SOX2, Ki67, LC3B, and cleaved caspase-3 in xenograft tumors harvested at the end point of the experiment. The results showed that CDH1 expression was obviously decreased, whereas SOX2 and Ki67 expression was increased in FaDu-CDH1i tumors (Fig. 7C), confirming the reliability of the xenograft model. In addition, afatinib induced significantly less LC3B and more cleaved caspase-3 in FaDu-CDH1i xenograft tumors compared to control (Fig. 7C). In summary, our results provide in vivo evidence that afatinib stimulated weaker autophagy in CDH1-silenced tumors, which has the potential to be the intrinsic driving factor for selectively eradicating stem-like HNSCC cells.

## Discussion

Emerging studies have documented that afatinib, a novel and irreversible pan-EGFR inhibitor, effectively induces autophagy in lung cancer cells [15, 16]. However, the molecular mechanism and biological function of afatinib-induced autophagy in HNSCC remains largely unknown. Herein, the primary purpose of our investigation was focused on: 1) ascertaining whether afatinib induces autophagy in HNSCC cells; 2) illuminating the molecular mechanism of afatinib-triggered autophagy; 3) elaborating, for the first time, the relationship between afatinib-induced autophagy and apoptosis in HNSCC cells, as well as stem-like HNSCC cells.

Autophagy is a highly conserved catabolic and homeostatic cellular process by which defective cytoplasmic cargoes are engulfed into autophagosomes, degraded in lysosomes, and recycled to maintain cellular metabolism in the face of an adverse physiological environment [21, 34]. In the current study, we demonstrated that afatinib induced transformation of LC3B-II and accumulation of EGFP-LC3B spots in HNSCC cells. P62, an important autophagy substrate, was also markedly downregulated following afatinib treatment. Moreover, pharmacological or genetic inhibition of autophagy resulted in a significant blockade to afatinib-induced autophagy flux. Taken together, these results demonstrated that afatinib stimulated autophagy in human HNSCC cells.

Although a broad spectrum of signaling pathways function to modulate autophagy, the mTOR pathway is characterized as the master negative regulator in autophagy initiation [35]. In our previous study, we reported that mTORC1 was inactivated by afatinib treatment [13]. To further determine whether afatinib-induced autophagy was attributed to mTORC1 suppression, rapamycin was used to imitate the suppressive effect of afatinib on mTORC1, and the conversion of LC3B-II was upregulated in a dose-dependent fashion. In addition, co-treatment with rapamycin strengthened afatinib-induced LC3B-II transformation, suggesting that mTOR inhibition was in fact responsible for afatinib-induced autophagy. Previous studies have demonstrated that the TSC1/TSC2 complex, the pivotal negative modulator of mTOR, was tightly involved in autophagy regulation [36]. We found that TSC1 and TSC2 levels were dramatically elevated following afatinib treatment in HNSCC cells. However, silencing TSC1 (but not TSC2) impaired mTOR inactivation and LC3B-II transformation, indicating that only TSC1 participated in afatinib-induced mTORC1 suppression and subsequent autophagy.

REDD1 is a stress-responsive gene that plays a pivotal role in the regulation of TSC1/2-associated mTORC1 suppression [37, 38]. In the present study, we demonstrated that REDD1 was elevated following afatinib exposure, and found that REDD1 knockdown dramatically impaired TSC1 upregulation, as well as mTOR inactivation. In parallel, we observed a decrease of LC3B-II conversion, suggesting that the afatinib-induced increase in REDD1 was required for TSC1/mTOR-mediated autophagy. ROS have been identified as important inducers for REDD1 expression, and have crucial functions in the stimulation of autophagy [39, 40]. In the present study, we discovered that ROS levels increased after afatinib treatment. Hampering ROS relieved afatinib-induced the upregulation of REDD1 and TSC1, and the inactivation of mTOR. Meanwhile, afatinib-induced LC3B-II conversion was concordantly attenuated without ROS. In conclusion, our data suggested that afatinib stimulated REDD1-TSC1 signaling via ROS accumulation, which in turn inhibited mTORC1 to promote autophagy in HNSCC cells.

Autophagy works as a double-edged sword in cancer, acting as either a survival mechanism to promote tumor progression or as a tumor suppressor by eliciting cell death in response to different physiological settings [41]. Lee et al. have discovered that afatinib-triggered autophagy significantly facilitated SAHA-induced apoptosis in EGFR T790M-mutated lung cancer [16]. Furthermore, the synergetic lethal effect of co-treatment with afatinib and adriamycin may be attributed to apoptosis as well as autophagy promoted by afatinib [17]. However, we and others proposed a conflicting view that afatinib induced protective autophagy in cancer cells. Hu et al. demonstrated that blocking autophagy utilizing the autophagy inhibitors CQ and 3-MA significantly strengthened the sensitivity of lung adenocarcinoma cells to afatinib-induced apoptosis, which was confirmed by in vitro and in vivo assays [15]. Recently, it has been reported that autophagy contributed to afatinib resistance in pancreatic ductal adenocarcinoma (PDAC). Knocking-down Beclin-1 to block autophagy significantly decreased the half-maximal inhibitory concentration of afatinib in afatinib-resistant cells [14]. Importantly, we found that inhibition of autophagy, either by combined treatment with autophagy inhibitors or by Atg5 siRNA transfection, increased afatinib-induced apoptosis in HNSCC cells. Collectively, these findings suggested that the function of afatinib-induced autophagy varied, depending on diverse drug combinations and cancer types. Additional studies are needed to better elucidate the mechanism of afatinib-induced autophagy in HNSCC.

Afatinib exhibits an improved treatment benefit in R/M HNSCC patients when compared to traditional second-line chemotherapy agents, as assessed by phase III clinical trials [7, 8]. Because stem cells are largely responsible for tumor recurrence, metastasis, and chemoresistance [42], we hypothesized that afatinib selectively eradicates stem-like HNSCC cells to confer treatment advantage to R/M HNSCC patients. Our findings demonstrated that afatinib induced more apoptosis, coupled with less autophagy, in CDH1-depleted HNSCC cells in vitro and in vivo experiments. These data showed that afatinib preferentially targeted stem-like HNSCC cells. Thus, our results suggested that blocking autophagy has the potential to serve as a new strategy for eliminating stem-like HNSCC cells. Furthermore, these findings suggested the potential for a novel clinical application using afatinib in the treatment of HNSCC.

**Fig. 8:**
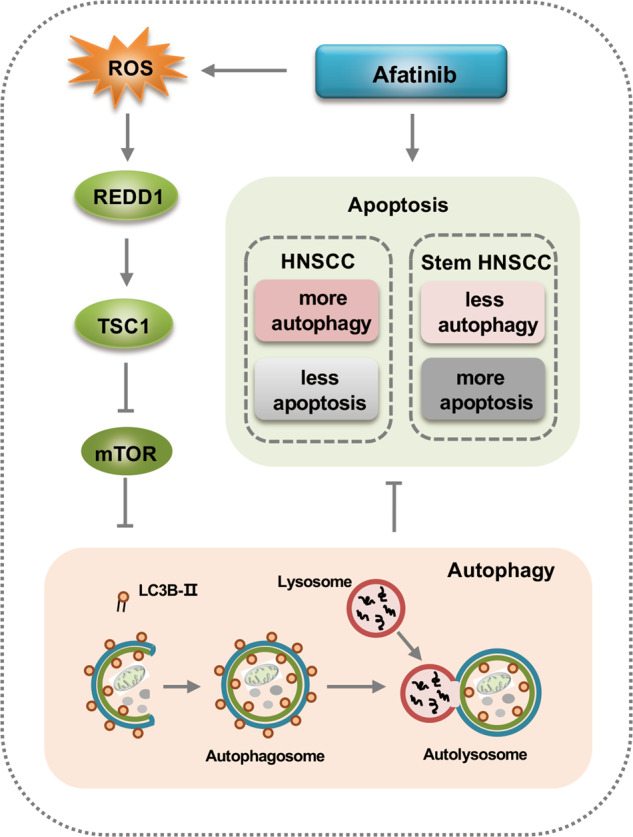
Schematic illustration of the mechanism and function of afatinib-induced autophagy. Afatinib induces pro-survival autophagy in HNSCC via the ROS-REDD1-TSC1-mTOR axis. Importantly, afatinib preferentially eliminates cancer stem-like HNSCC cells that may attribute to weaker autophagy induction. This suggests that combined treatment with afatinib and autophagy inhibitors might serve as a promising strategy for improving the clinical outcome of afatinib in HNSCC treatment.

In summary, we demonstrated that afatinib induced antiapoptotic autophagy in HNSCC through ROS-REDD1-TSC1-mediated mTOR suppression. Importantly, the present study showed that afatinib preferentially eradicated stem-like HNSCC cells by inducing severe apoptosis and only weakly activating an autophagic response (Fig. 8). Therefore, our results suggested that combined treatment of afatinib with autophagy inhibitors might serve as a promising strategy for improving the clinical outcome of afatinib in HNSCC treatment.

## Supplementary information

Supplementary Figure 1

Supplementary Figure 2

Supplementary Figure 3

Supplementary Figure 4

Supplementary Figure 5

Supplementary Materials and Methods and Figure Legends

Supplementary Tables
