# Supplementary figures and images for: Afatinib induces pro-survival autophagy and increases sensitivity to apoptosis in stem-like HNSCC cells
Source: Cell Death Dis. 2021 Jul 22;12(8):728. doi: 10.1038/s41419-021-04011-0 (PMC8298552; doi:10.1038/s41419-021-04011-0)

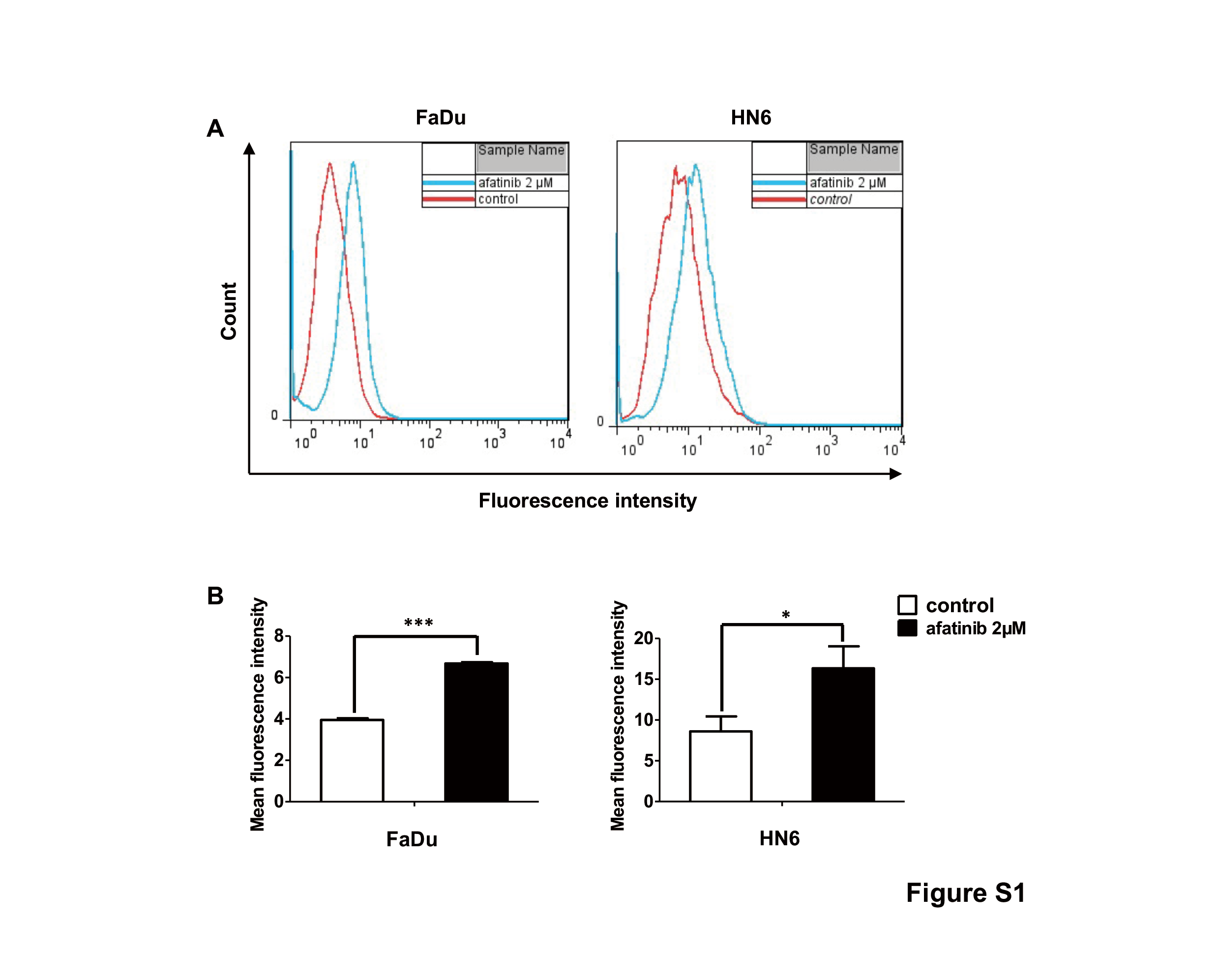

Supplement: Supplementary file 1 — Supplementary Figure 1 [file 41419_2021_4011_MOESM1_ESM.png]

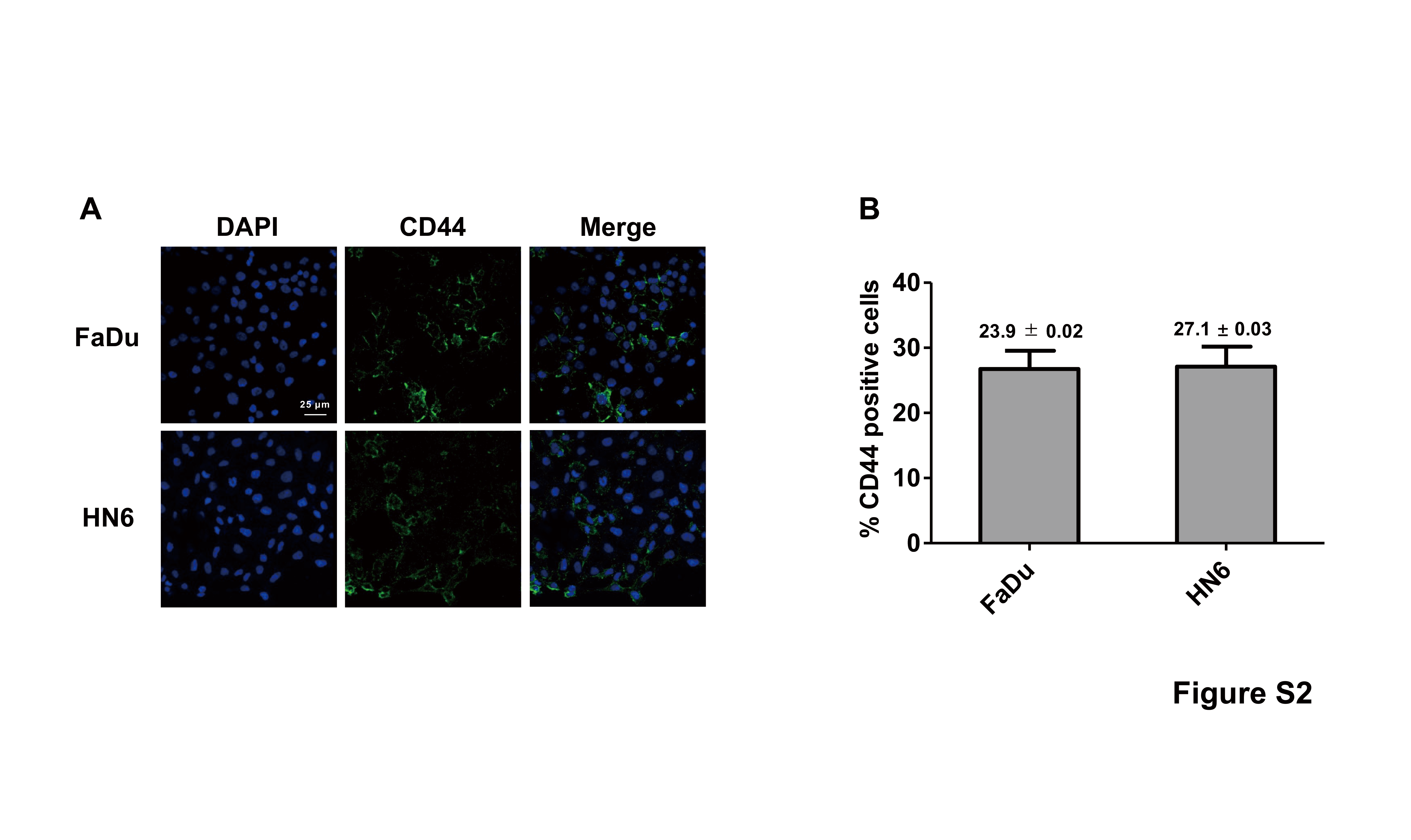

Supplement: Supplementary file 2 — Supplementary Figure 2 [file 41419_2021_4011_MOESM2_ESM.png]

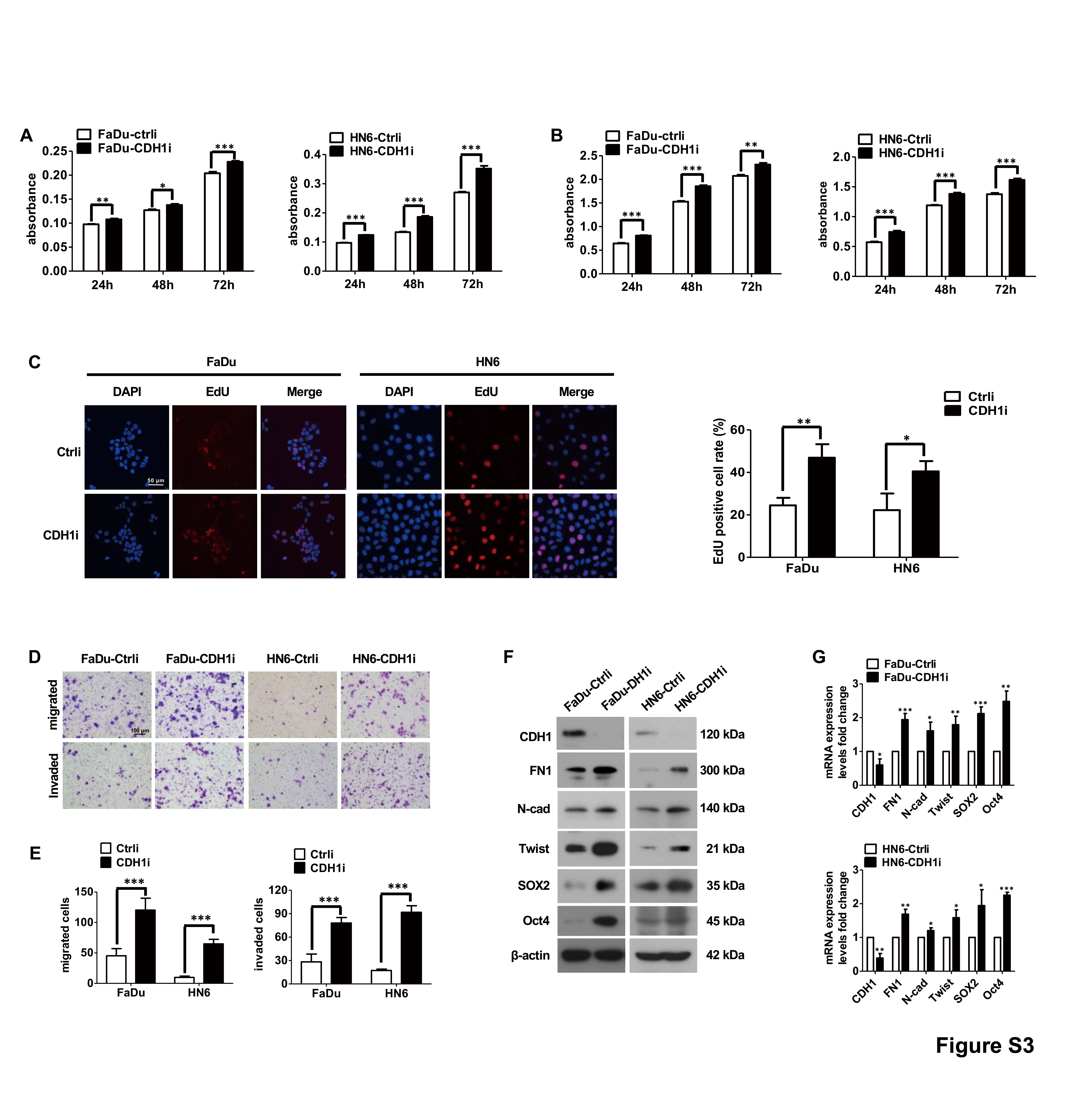

Supplement: Supplementary file 3 — Supplementary Figure 3 [file 41419_2021_4011_MOESM3_ESM.png]

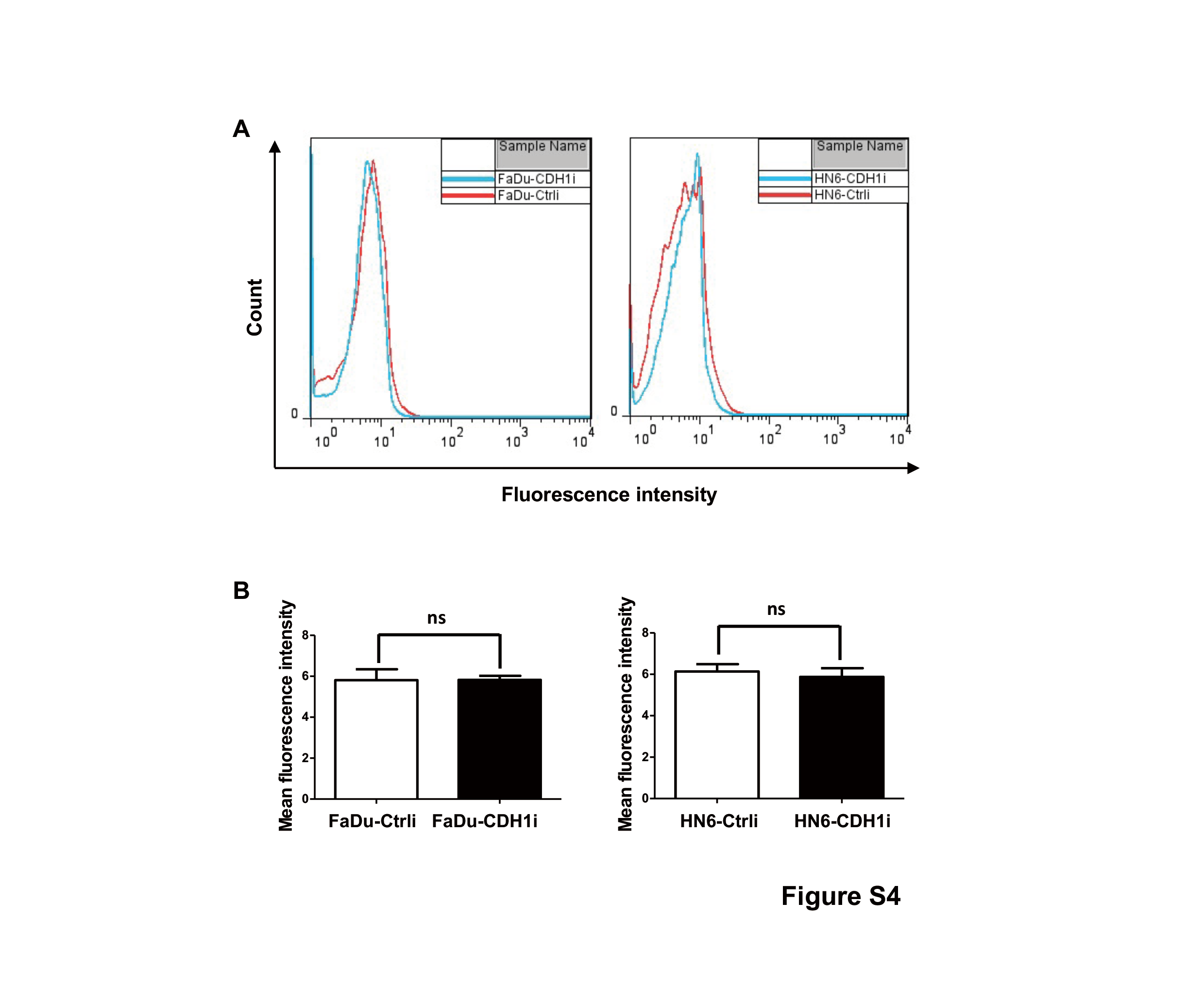

Supplement: Supplementary file 4 — Supplementary Figure 4 [file 41419_2021_4011_MOESM4_ESM.png]

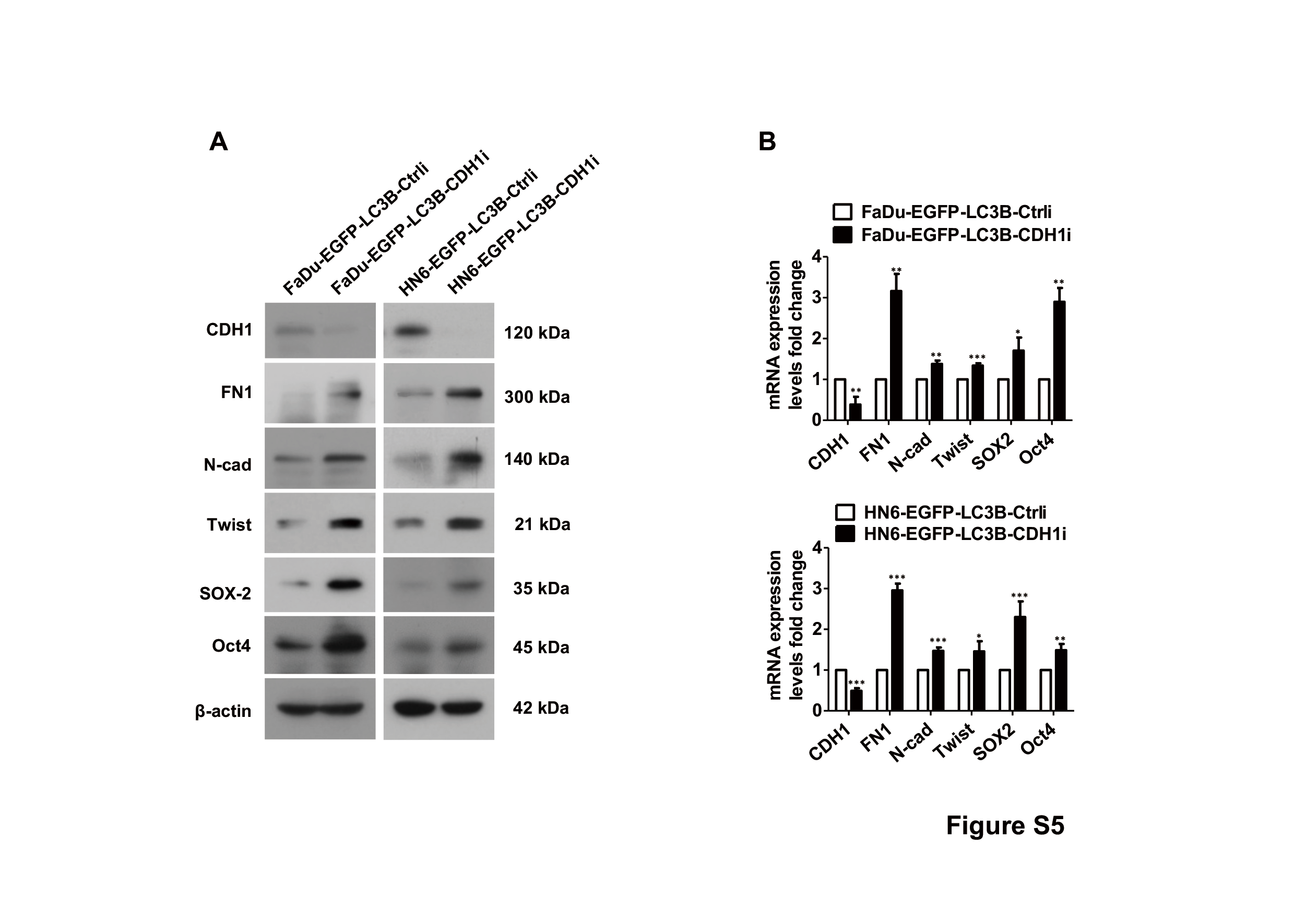

Supplement: Supplementary file 5 — Supplementary Figure 5 [file 41419_2021_4011_MOESM5_ESM.png]
