# Supplementary material for: Afatinib induces pro-survival autophagy and increases sensitivity to apoptosis in stem-like HNSCC cells
Source: Cell Death Dis. 2021 Jul 22;12(8):728. doi: 10.1038/s41419-021-04011-0 (PMC8298552; doi:10.1038/s41419-021-04011-0)
Supplement: Supplementary file 6 — Supplementary Materials and Methods and Figure Legends [file 41419_2021_4011_MOESM6_ESM.docx]

**Supplementary Materials and Methods**

**Immunofluorescence**

Cells were seeded in 48-well plates with cell slides (4.2×10^4^ cells/well for FaDu; 2.1×10^4^ cells/well for HN6). After 48 h, cells were fixed with 4% paraformaldehyde for 30 min and blocked with 3% BSA for 1 h at room temperature. Then, cells were incubated with anti-human CD44 antibody pre-conjugated with FITC (Thermo Fisher Scientific, Waltham, MA, USA) for 30 min, followed by incubation with DAPI for nuclear counterstaining. Fluorescent images were captured utilizing a laser scanning confocal microscope (Leica, Germany).

**Supplementary Figure Legends**

**Figure S1. Afatinib causes ROS accumulation in HNSCC cells. A**, **B** FaDu and HN6 cells were treated with 2 μM afatinib for 24 h, followed by staining with DCFH-DA for 20 min. The cellular ROS levels were evaluated by flow cytometry analysis (**A**). The value is presented as the mean ± SD of three independent experiments (**B**). ^*^*p* < 0.05, ^***^*p* < 0.001.

**Figure S2. The expression of the putative mesenchymal stem marker CD44 in FaDu and HN6 cell lines. A** Representative laser scanning confocal microscope images with CD44 (green) and DAPI (blue) of FaDu and HN6 cells. Scale bar, 25 µm. **B** The percentage of cells with CD44 expression (CD44-positive cells/DAPI-labeled cells) was quantified.

**Figure S3. FaDu and HN6 cells with CDH1 depletion display stem-like cancer cell characteristics. A**, **B** FaDu and HN6 cells with CDH1 depletion and corresponding control cells were seeded in 96-well plates for 24 h, 48 h, and 72 h respectively. Cell growth was assessed with the SRB assay (**A**), and cell proliferation was evaluated using the CCK8 assay (**B**). **C** FaDu and HN6 cells with CDH1 depletion and control cells were cultured in 48-well plates for 24 h. Cell proliferation ability was detected by EdU staining. Scale bar, 50 µm. **D**, **E** Images of migrated and invaded cells were captured (**D**) and quantitative analysis of migrated and invaded cells was performed (**E**). Scale bar, 100 µm. **F, G** Western blot (**F**) and RT-qPCR (**G**) analysis of CDH1, FN1, N-cad, Twist, SOX2, and Oct4 expression in FaDu and HN6 cells with CDH1 depletion and in control cells. Data are presented as the mean ± SD from three independent experiments. ^*^*p* < 0.05, ^**^*p* < 0.01, ^***^*p* < 0.001.

**Figure S4. No difference in ROS levels in FaDu and HN6 cells with CDH1 depletion compared to control cells. A**, **B** After 24 h of culture, FaDu and HN6 cells with CDH1 depletion as well as corresponding control cells were stained with DCFH-DA for 20 min and the cellular ROS levels were evaluated by flow cytometry analysis (**A**). The value is presented as the mean ± SD of three independent experiments (**B**). ns: no significance.

**Figure S5. Upregulation of mesenchymal and stem cell markers in FaDu-EGFP-LC3B and HN6-EGFP-LC3B cells with CDH1 depletion. A, B** Western blot (**A**) and RT-qPCR (**B**) analysis of CDH1, FN1, N-cad, Twist, SOX2, and Oct4 expression in FaDu-EGFP-LC3B and HN6-EGFP-LC3B cells with CDH1 depletion and in control cells.
