# Supplementary material for: Afatinib induces pro-survival autophagy and increases sensitivity to apoptosis in stem-like HNSCC cells
Source: Cell Death Dis. 2021 Jul 22;12(8):728. doi: 10.1038/s41419-021-04011-0 (PMC8298552; doi:10.1038/s41419-021-04011-0)
Supplement: Supplementary file 7 — Supplementary Tables [file 41419_2021_4011_MOESM7_ESM.docx]

**Supplementary Table 1. RT-qPCR primers**

| **Gene** | **Forward primer** | **Reverse primer** |
| --- | --- | --- |
| *CDH1* | TGCCCAGAAAATGAAAAAGG | GTGTATGTGGCAATGCGTTC |
| *FN1* | CAGTGGGAGACCTCGAGAAG | TCCCTCGGAACATCAGAAAC |
| *N-cad* | ACAGTGGCCACCTACAAAGG | CCGAGATGGGGTTGATAATG |
| *Twist* | GGAGTCCGCAGTCTTACGAG | TCTGGAGGACCTGGTAGAGG |
| *SOX2* | TGGACAGTTACGCGCACAT | CGAGTAGGACATGCTGTAGGT |
| *Oct4* | CTTGAATCCCGAATGGAAAGGG | CCTTCCCAAATAGAACCCCCA |
| *β-actin* | CCAACCGCGAGAAGATGA | CCAGAGGCGTACAGGGATAG |

**Supplementary Table 2. The IC_50_ value of afatinib in FaDu, HN6, and CAL-27 cell lines.**

| Cell lines | FaDu | HN6 | CAL-27 |
| --- | --- | --- | --- |
| IC_50_ (μM) | 2.742 | 1.296 | 4.997 |

**Supplementary Table 3. Basal expression of Beclin-1, LC3B-II, p62, and p-mTOR in FaDu, HN6, and CAL-27 cell lines.**

|  | The intensity of tested molecules | | | |
| --- | --- | --- | --- | --- |
| Cell lines | Beclin-1 | LC3B-II | p62 | p-mTOR |
| CAL-27 | 1.00 | 1.00 | 1.00 | 1.00 |
| FaDu | 0.51 | 0.31 | 1.37 | 0.77 |
| HN6 | 0.36 | 0.33 | 1.71 | 0.68 |
